# Non-small cell lung cancer cells and concomitant cancer therapy induce a resistance-promoting phenotype of tumor-associated mesenchymal stem cells

**DOI:** 10.3389/fonc.2024.1406268

**Published:** 2024-07-01

**Authors:** Hanna Sentek, Annika Braun, Bettina Budeus, Diana Klein

**Affiliations:** Institute of Cell Biology (Cancer Research), University of Duisburg-Essen, University Hospital, Essen, Germany

**Keywords:** mesenchymal stem cells, adventitia, senescence, SASP, lung cancer, NSCLC, radiotherapy, resistance

## Abstract

**Introduction:**

The tumor microenvironment gained attraction over the last decades as stromal cells significantly impact on tumor development, progression and metastasis, and immune evasion as well as on cancer therapy resistance. We previously reported that lung-resident mesenchymal stem cells (MSCs) were mobilized and activated in non-small cell lung cancer (NSCLC) progression and could even mediate radiation resistance in co-cultured NSCLC cells.

**Methods:**

We investigated how MSCs were affected by NSCLC cells in combination with cancer (radiation) therapy in indirect co-cultures using tumor-conditioned medium and Transwells or direct three-dimensional NSCLC–MSC spheroid co-cultures in order to unravel the resistance-mediating action of tumor-associated MSCs.

**Results:**

Although no obvious phenotypic and functional alterations in MSCs following NSCLC co-culture could be observed, MSC senescence was induced following co-applied radiotherapy (RT). Global gene expression profiling, in combination with gene set enrichment analysis upon treatment, was used to confirm the senescent phenotype of irradiated MSC and to reveal relevant senescence-associated secretory phenotype (SASP) factors that could meditate NSCLC RT resistance. We identified senescent tumor-associated MSC-derived serine proteinase inhibitor (serpin) E1/PAI1 as potential SASP factor mediating NSCLC progression and RT resistance.

**Discussion:**

Specified intra-tumor–stroma interactions and cell type-specific pro-tumorigenic functions could not only improve lung cancer classification but could even be used for a more precise profiling of individual patients, finally paving an additional way for the discovery of potential drug targets for NSCLC patients.

## 1 Introduction

Early-onset tracheal, bronchial, and lung cancer remains one of the cancers with the highest incidence worldwide (with the second largest number of 2.24 million new cases, accounting for 11.4% of all cancers in 2020) while remaining as the leading cause of cancer-related deaths (1.8 million deaths, 18%), numbers that are expected in increasing trends (1, 2). Although treatment options including surgery, chemotherapy, radiation therapy (RT), and targeted therapy, most often used as combination therapies, are substantially improved nowadays (e.g., minimally invasive techniques, stereotactic ablative radiotherapy; new targeted therapies and immunotherapies), only around 20% of all lung cancer patients are alive 5 years or later after diagnosis (3, 4). Lung cancer is a highly heterogeneous disease. Generally, lung cancer is divided into small cell lung carcinoma (SCLC) and the more frequently occurring non-small cell lung carcinoma (NSCLC), in which the histological subtypes lung adenocarcinoma (adeno CA), squamous cell carcinoma (squamous), and large cell lung cancer are distinguished (5). Besides the malignant epithelial cells themselves, recruited non-epithelial stromal cells, ostensibly normal cells at the beginning, acquire tumor-promoting phenotypes upon crosstalk with cancer cells, and this neoplastic stroma decisively impacts on invasive growth and metastasis, determining disease progression and therapy resistance (6, 7), particularly in lung cancer (8, 9). Besides infiltrating immune cells and vascular cells regulating immune cell homing as well as oxygen and nutrient delivery, numerous phenotypically similar but functionally different mesodermal cells, including activated fibroblasts, namely myo- and cancer-associated fibroblasts, and tumor-associated mesenchymal stem cells (MSCs), also designated as multipotent stromal cells, comprise the tumor stroma (8–13). We previously reported that lung-resident MSCs, usually localized within the vascular adventitia as stem cell niche, were mobilized and activated in NSCLC progression (14). Using three-dimensional spheroidal tumor-stroma co-cultures, we further showed that MSCs (similar to fibroblasts) mediate radiation resistance in co-cultured lung carcinoma cells (14). As these findings might have implications for the rational design of additional therapeutic approaches, we investigated here how NSCLC cells impact on MSC cellular features and potentially “educate” initially normal MSCs to become tumor-promoting MSCs.

## 2 Materials and methods

### 2.1 Cell cultures

The NSCLC cell lines NCI-H460 and A549 (both from ATCC; Manassas, VA, USA) were cultured in RPMI 1640 and DMEM media (Gibco, Thermo Fisher Scientific, Waltham, MA, USA), each supplemented with 10% fetal calf serum (FCS) and 100 U penicillin/streptomycin (Sigma-Aldrich, St. Louis, MO, USA). The cells were cultured under standard cell culture conditions at 37°C and 5% CO_2_. All cells were routinely tested for mycoplasma contamination (every month) and periodically authenticated by STR profiling (if necessary, no later than yearly). Primary adventitial MSCs (VW-MSCs) were routinely isolated from human internal thoracic arteries obtained during surgery (fragments) as described before (15–17) and cultivated on plastic cell culture plates using complete human MSC-GM media (PromoCell, Heidelberg, Germany). All experiments were performed in strict accordance with local ethical and biohazard regulations and were approved by the local ethic committee. Informed consent (written form, nos. 10–4363 and 17–7454-BO) was obtained from the Ethik-Kommission, University Medical Faculty, Essen, Germany (14, 15, 18, 19). Irradiations of cell cultures were performed using an Isovolt-320-X-ray machine (Seifert-Pantak) at 320 kV, 10 mA, and a 1.65-mm aluminum filter at a distance of 50 cm (at room temperature). The dose rate was approximately 3 Gy/min with the energy of the tube at 90 kV (~ 45 keV X-rays). For the spheroid culture, the cells were cultured alone or in indicated combinations with hanging drops for 24 h (ratio 1/1) and as previously described (14, 20). Afterwards, spheroids were plated in growth factor-reduced Matrigel (Corning, NY, USA) [dilution: 1/2 with normal growth medium (NGM)], and irradiated 2 h later. Pictures were taken directly and at 48 h (and 7 days) after treatment at ×10 magnification. The spheroid size was measured and calculated using ImageJ software as previously described (14, 20). For the detection of cell death, the spheroids were incubated thereafter for additional 15 min with 50 µg/mL propidium iodide and 1 µg/mL Hoechst 33342 (ThermoFisher, Waltham, MA, USA) for nuclei staining and analyzed by phase-contrast and fluorescent microscopy using ZEISS Axio Observer (Carl Zeiss, Oberkochen, Germany). For the generation of conditioned media, respective cells were cultured in NGM until 80% confluence. The media were replaced, and the cells were cultured for an additional 48 h before the collection of media. The supernatant was harvested and centrifuged to separate dead cells. Control media were generated by incubating the same NGM without cells. Conditioned media were used as 1/2 mixture with NGM (14, 21). For the indirect (Transwell) co-culture, MSCs were plated in six-well plastic dishes. Transwells were added on the top of the cultures, and NSCLC cells were plated into each Transwell at the same cell concentration (ratio 1/1) as previously described (22).

### 2.2 Colony‐forming unit and clonogenic survival assay

Cells were plated at low densities (100–1,000 cells per well; triplicates), treated as indicated, and after additional 10 days of culture, the cells were washed with PBS, fixed with 4% (w/v) paraformaldehyde, and subsequently stained with 0.05% Coomassie Brilliant Blue. The colonies (≥50 cells/colony) were counted, and plating efficiencies (number of colonies observed/number of cells plated) were calculated (14, 16, 23). For clonogenic survival, plated cells were additionally irradiated with the indicated doses. After an additional 10 days, stained colonies were counted, and plating efficiencies and survival rates were calculated. Survival curves were established by plotting the log of the surviving fraction (20, 21).

### 2.3 Trilineage differentiation assay

Differentiation of cultivated MSCs into adipocytes, chondrocytes, and osteocytes was done using ready-to-use differentiation media from PromoCell (Heidelberg, Germany; Chondrogenic: C-28012; Osteogenic: C-28013; Adipogenic Differentiation Medium: C-28016) according to the manufacturer’s instructions. Adipogenic differentiation was verified using oil red staining, chondrogenic differentiation was verified using Alcian Blue staining solution (1% w/v Alcian Blue in acetic acid, pH 2.5), and osteogenic differentiation was verified using NBT (nitro-blue tetrazolium chloride) and BCIP (5-bromo-4-chloro-3’-indolyphosphate p-toluidine salt) staining (Sigma) for alkaline phosphatase activity (14, 18).

### 2.4 Cellular viability, proliferation, and migration analyses

For cell viability measurements, WST-1 (water-soluble tetrazolium salt; Sigma-Aldrich) was added (1/10) to respective cultures, and colorimetric changes were measured at 450 nm (optical density; OD) at the indicated time points as previously described (24, 25). Cellular proliferations were determined by crystal violet staining (0.1% crystal violet in PBS; Carl Roth, Karlsruhe, Germany) of PBS-washed and glutaraldehyde-fixed cells. Following Triton X-100 dye release, the dye concentrations were measured spectrophotometrically at 540 nm. For extracellular flux analysis, cells were plated on XF96 microplates (Seahorse Bioscience/Agilent Technologies, Santa Clara, CA, USA) according to the manufacturer’s instruction and as previously described (24, 25). For Mito Stress Test (Seahorse Bioscience), at 48 h post-treatment, the medium was changed to XF base medium, supplemented with 1 mM pyruvate, 2 mM glutamine, and 10 mM glucose, and incubated for 1 h at 37°C in a CO_2_-free incubator. Mitochondrial oxidative phosphorylation on the basis of the oxygen consumption rate (OCR) and glycolysis by analyzing the extracellular acidification rate (ECAR) were estimated following oligomycin (1 µM), carbonyl-cyanide-p-trifluoromethoxyphenylhydrazone (FCCP) (1 µM), and rotenone (0.5 µM) and antimycin A (0.5 µM) treatment at the indicated time points using Seahorse XFe 96 Analyzer. Hoechst 33342 (10 µg/mL, Thermo Fisher Scientific; Waltham, MA, USA) was used for individual normalization to DNA. Data were analyzed using Wave 2.6 software (Seahorse Bioscience). Cellular migrations were investigated via time lapse microscopy for the indicated time following radiation treatment as previously described (23, 24). Briefly, the cells were grown to confluence and irradiated, and a thin wound was introduced by scratching with a 10 µL pipette tip. Wound closure was determined for the different treatments by measuring the migration distance using ImageJ.

### 2.5 Flow cytometry

Flow cytometric measurements were performed on a BD LSRII flow cytometer using FACS DIVA software (BD Bioscience, Franklin Lakes, NJ, USA). Cell cycle phases were analyzed at the indicated time points post-RT using respectively harvested cells (by trypsinization) in combination with Nicoletti staining solution [50 μg/mL propidium iodide (PI), 0.1% sodium citrate (w/v), and 0.05% Triton X-100 (v/v) in PBS] (30-min incubation at room temperature prior analyses).

### 2.6 Western blotting

The generation of whole cell lysates was carried out by scraping cells off into ice-cold RIPA buffer (150 mmol/L NaCl, 1% NP40, 0.5% sodium-deoxycholate, 0.1% sodium-dodecylsulfate, 50 mmol/L Tris/HCl, pH 8, 10 mmol/L NaF, 1 mmol/L Na3VO4) supplemented with protease-inhibitor cocktail (Roche). After two to three freeze-and-thaw cycles, the protein content of the lysates was measured by using Bio-Rad DC™ Protein Assay. Then, 50–100 µg of total proteins were used for SDS-PAGE electrophoresis as previously described (24, 25). Representative blots from at least three independent experiments were shown.

### 2.7 RNA sequencing analysis

Total RNA was isolated from cultures following sham (Ctrl, 0 Gy) or 10-Gy irradiations at 96 h post-RT. The concentration and quality of RNA were measured with Qubit (Invitrogen, Waltham, MA, USA) and Agilent Bioanalyzer DNA HS (Agilent, Santa Clara, CA USA). Library preparation was performed with Lexogens QuantSeq 3′ mRNA-Seq Library Prep Kit FWD and sequenced on a NextSeq500 (Illumina, San Diego, CA, USA). The sequences were trimmed with TrimGalore (26) with standard settings and aligned with hisat2 (27) to hg38 with standard settings. Statistical analysis was performed with R [v. 4.3.1, R Core Team (2023). R: A language and environment for statistical computing. R Foundation for Statistical Computing, Vienna, Austria; URL: https://www.R-project.org/.] using the R-packages DESeq2 (28) for main DEG calculations, ComplexHeatmap (29) for heatmaps, umap (30) for dimensionality reduction, fgsea (31) for gene set enrichment analysis, and EnhancedVolcano (https://github.com/kevinblighe/EnhancedVolcano) for volcano plots as previously described (32). The accession number for the RNAseq data reported in this article is GSE248107 (GEO repository).

### 2.8 Kaplan–Meier plotter

The prognostic significance of mRNA expression levels of SERPINE1 and MMP2 in NSCLC were evaluated using the Kaplan–Meier plotter (www.kmplot.com) (33) as previously described (14).

### 2.9 Statistical analysis

If not otherwise indicated (*n* = biological replicates), data were obtained from at least three independent experiments. Data were presented as mean values ± SD or SEM. Data analyses were performed by indicated one- or two-way ANOVA, followed by Tukey’s multiple-comparison post-tests or by unpaired (two-tailed) *t*-tests using Prism 8.0 software (GraphPad, La Jolla, CA, USA). Statistical significance was set at the level of *p* ≤ 0.05.

## 3 Results

### 3.1 Rather than MSC–NSCLC interactions, concurrent RT treatment alters the phenotype of MSCs

**Figure 1:**
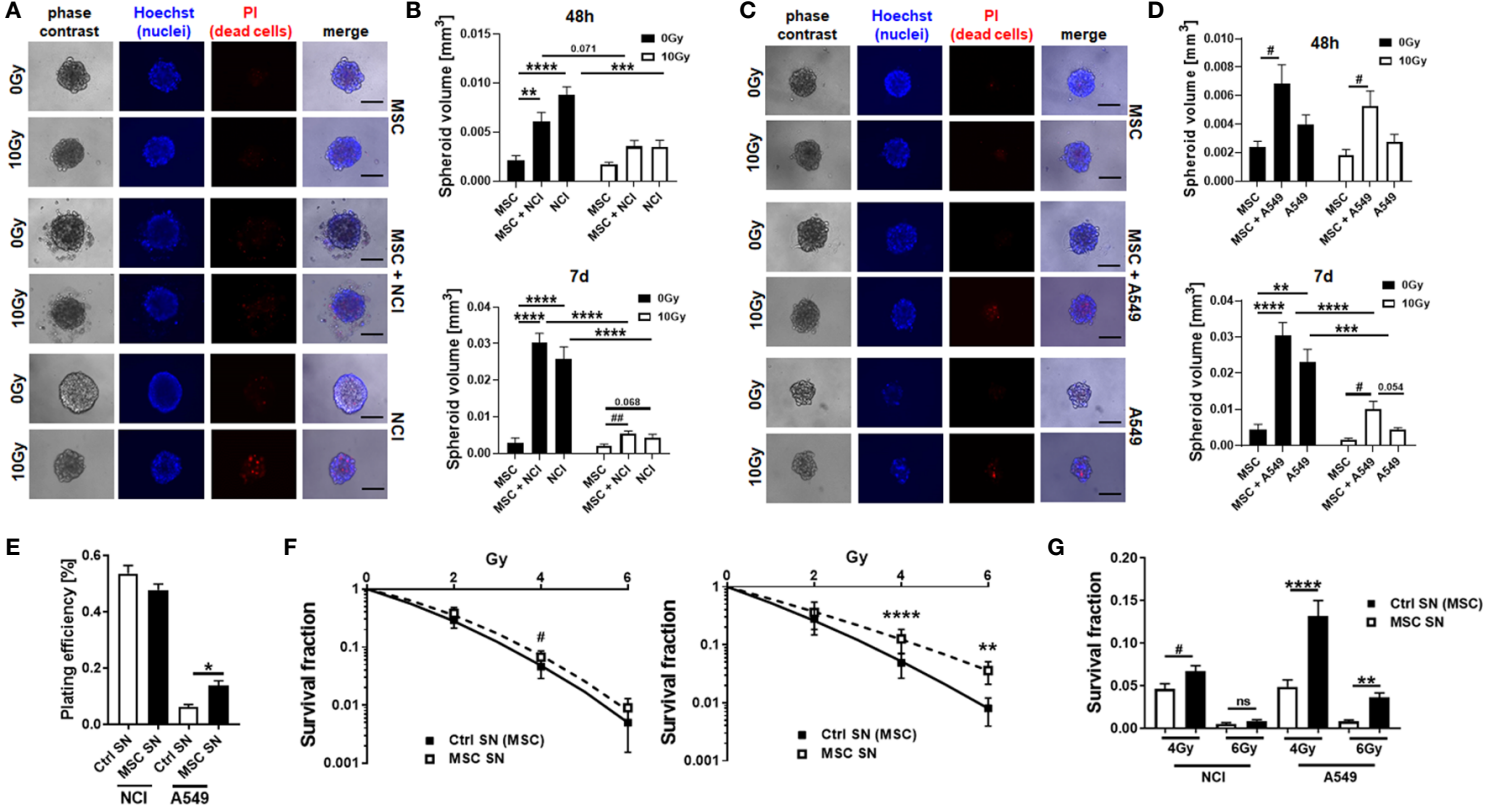
(TA-) MSCs mediate RT resistance in directly co‐cultured MSCs–NSCLC spheroids. NCI-H460 **(A, B)** and A549 **(C, D)** lung cancer were cultured alone or co-cultured with VW-MSCs in hanging drops for 24 h. After the formation of spheroids, cells were plated in GFR-Matrigel mixed with normal growth medium (1:2, v/v) and left untreated or irradiated at 10 Gy. Spheroids generated from VW-MSCs only were additionally included. Cell death was analyzed afterwards (48-h time point) by fluorescence microscopy using propidium iodide **(A, C)**. Hoechst 83342 was used for nuclei staining. Representative phase-contrast images and simultaneously recorded fluorescent photographs from three individual experiments are shown. The scale bar represents 25 µm. Spheroids’ growth was measured after an additional 48 h and 7 days of cultivation, and the respective volumes were calculated **(B, D)**. The graphs depict the measurements from five to eight independent experiments where at least 10 spheroids per condition were measured. **p < 0.01; ***p < 0.005; ****p < 0.001 by two-way ANOVA followed by *post hoc* Tukey’s comparison test and additionally by unpaired (two-tailed) *t*-tests depicted as ^#^ *p* ≤ 0.05; ^##^ *p* ≤ 0.01. **(E–G)** Lung cancer cells were plated at low density (CFU assay), irradiated with the indicated doses (0, 2, 4, and 6 Gy), and further incubated for additional 10 days in conditioned media (SN) derived from cultured MSCs or control (Ctrl) SN. Quantification of grown (plating efficiency) and surviving colonies was performed after Coomassie Brilliant Blue staining. The graphs depict data from three to five independent experiments, each measured in *P*-value by one-way ANOVA, followed by *post hoc* Tukey’s multiple-comparison test: *P ≤ 0.05, **P ≤ 0.01, ****P ≤ 0.001 and additionally by unpaired (two-tailed) *t*-tests depicted as ^#^ *p* ≤ 0.05. ns, non significant.

**Figure 2:**
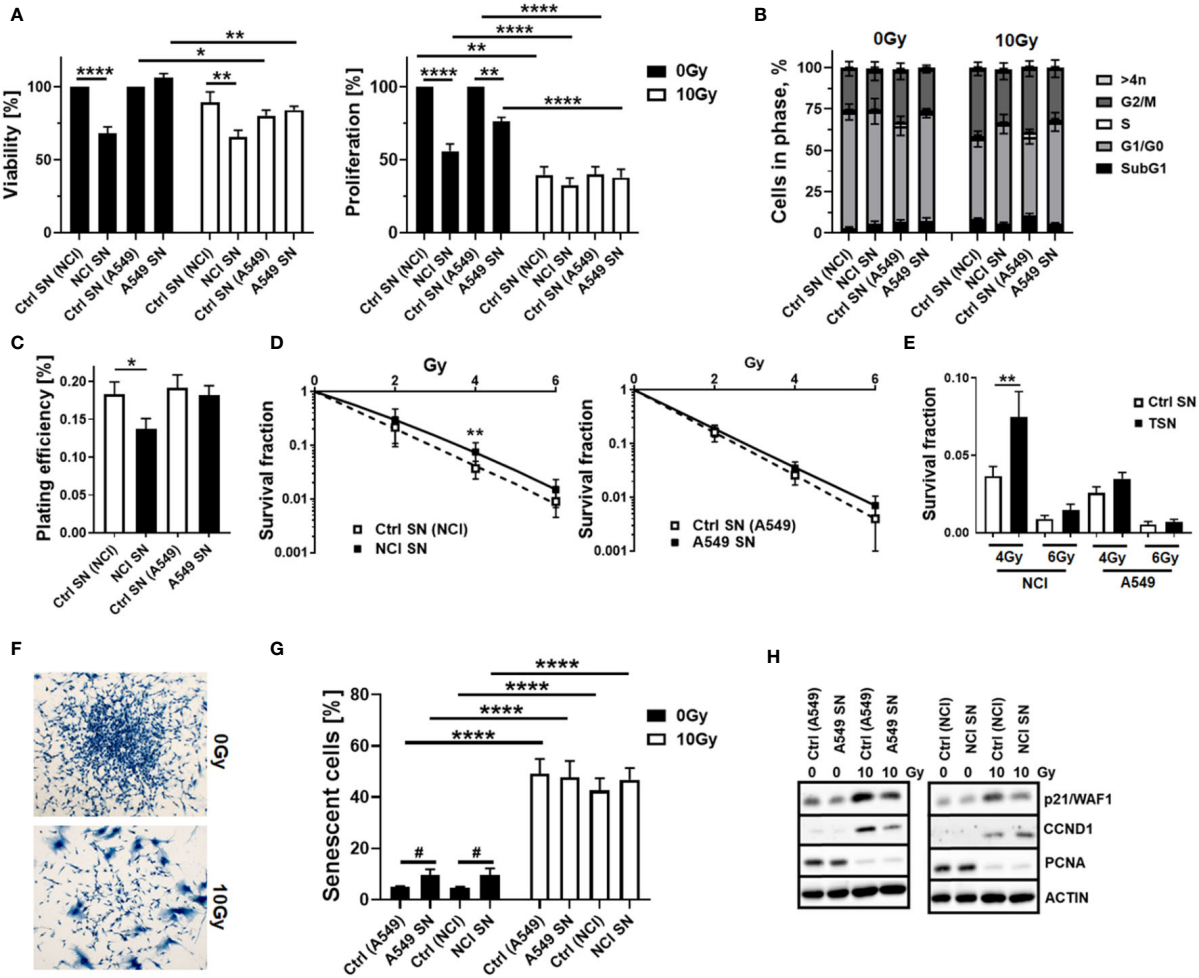
Tumor-secreted factors derived from NCI-H460 NSCLC cells reduced MSC viabilities and proliferation levels and fostered a more radioresistant MSC phenotype, while A549 NSCLC cell-derived factors did not affect MSC’s cellular features. Cellular features of cultured MSCs were analyzed after treatment with tumor-conditioned media (SN; derived from cultured NCI-H460 and A549 NSCLC cells) in combination with or without radiation treatment (RT; 10 Gy) after 96 h. **(A)** The viabilities of MSCs were analyzed using the WST-1 reagent, and the proliferation levels of MSCs were further determined using crystal violet staining. Estimated values were related to respective control SN (Ctrl SN) treatments (0 Gy; set as 100%). Data represent mean values ± SEM from four to five independent experiments measured in quadruplets each. *P*-values are indicated as follows: *p ≤ 0.05, **p ≤ 0.01, ****p ≤ 0.001 by two-way ANOVA with Tukey’s multiple-comparison test. **(B)** Cell cycle phases and apoptotic cells (subG1) were analyzed by flow cytometry. The graphs consist of data from five to eight individual experiments (with SEM). The statistically significant differences within G1/G0 [Ctrl (NCI) 0 Gy vs. Ctrl (NCI) 10 Gy: *p* ≤ 0.005), Ctrl (A549) 10 Gy vs. A549 SN 10 Gy: *p* ≤ 0.05], and G2/M phases [Ctrl (NCI) 0 Gy vs. Ctrl (NCI) 10 Gy: *p* ≤ 0.05] as estimated by two-way ANOVA with Tukey’s multiple-comparison test were not depicted. **(C)** The colony formation capacity was further evaluated by plating MSCs at low densities (CFU assay) in the presence or absence of NSCLC-derived factors, and **(D, E)** the clonogenic survival of MSCs was evaluated additionally following RT with the indicated doses (10 days post-treatment). The graphs depict data from three to five independent experiments, each measured in triplicate. *P*-values are indicated as *P ≤ 0.05 and **p ≤ 0.01 by one-way ANOVA, followed by *post hoc* Tukey’s multiple-comparison test. **(F)** Morphological alterations of MSCs in response to RT treatment were visualized following crystal violet staining. Representative photographs from control (Ctrl; 0 Gy) and RT (10 Gy)-treated MSCs are exemplarily shown. Magnification: ×10. **(G)** RT-induced senescence formation was analyzed by C12FDG staining prior to flow cytometry analyses 96 h post-treatment. The graphs depict data from four to eight independent experiments. *P*-value by two-way ANOVA, followed by *post hoc* Tukey’s multiple-comparison test: ****p ≤ 0.001 and additionally by unpaired (two-tailed) *t*-tests depicted as ^#^ *p* ≤ 0.05. **(H)** The expression levels of the indicated proteins were analyzed in whole protein lysates of cultured MSCs with or without radiation treatment (96 h after RT with 10 Gy) in the presence of the control or NSCLC-conditioned media (SN) using Western blot analysis. Representative blots from at least four independent experiments are shown.

As our previous studies strongly accounted for an involvement of local (tissue-resident) MSCs in the progression of NSCLC and particularly NSCLC cell resistance to RT (14), we extended respective experiments by including other NSCLC cells, namely, A549 adenocarcinoma cells. We first investigated how MSCs alter the RT response of lung cancer cells by using direct three-dimensional MSC-NSCLC spheroid co-cultures (embedded in growth factor-reduced Matrigel) in combination with radiation treatment (**Figure 1**). According to our previous observations (14), MSCs tend to reduce the growth of H460 cells upon direct co-culture, whereas upon RT the radiation-induced growth delay of H460 cells was limited when co-cultured with MSCs (**Figures 1A, B**). At the same time, the reduction of H460 spheroid growth upon RT was accompanied by the presence of tumor cells that were permeable for propidium iodide, thus showing RT-induced cell death, while the reduced growth delay of MSC-H460 spheroids following RT was not accompanied by increased cell death levels, indicating RT resistance (**Figures 1A, B**). In contrast, MSCs increased the growth of A549 cells upon direct co-culture, again with a limited growth delay following RT of MSC-A549 spheroid co-cultures, and reduced cell death levels, corroborating that MSCs were able to affect lung cancer cell proliferation and potentially mediate a more resistant phenotype (**Figures 1C, D**). Conformingly, although the plating efficiency was not affected (**Figure 1E**), MSC-derived factors (by using cell culture supernatants of VW-MSCs cultures) were able to increase the clonogenic survival of H460 cells following RT (**Figures 1F, G**). Moreover, MSC-derived factors were able to significantly increase the plating efficiency of A549 cells as well as the clonogenic survival of these cells following RT (**Figures 1E–G**). We next investigated how the MSCs were affected by NSCLC cells, particularly by NSCLC cell-derived factors. Therefore, cellular features of cultured MSCs were analyzed after treatment with tumor-conditioned media (SN; derived from cultured NCI-460 and A549 NSCLC cells) in combination with or without radiation treatment (RT; 10 Gy) after 96 h (**Figure 2**). Concerning the viabilities, conditioned media derived from NCI-H460 cells reduced the MSC viabilities under steady-state conditions, while RT only minimally reduced the cellular viabilities (**Figure 2A**, left diagram). In contrast, conditioned media derived from A549 cells did not affect the MSC’s viability nor in combination with RT. A more detailed analysis of the metabolic demands of cultured MSCs following tumor-secreted factor treatments with and without the combination of RT showed no obvious alterations of the metabolic potential under steady-state conditions, while respective reductions (in oxidative phosphorylation and glycolysis levels) were observed following RT (**Supplementary Figure S1**). The use of both conditioned media, however, significantly reduced the proliferation rates of cultured MSCs, an effect that was negligible in the context of reduced proliferations when combined with RT (**Figure 2A**, right diagram). The cell cycle analyses further revealed that NSCLC cell-derived factors did not obviously affect the cell cycle phase distribution at control (non-irradiated) conditions, while the reduced number of MSCs in G1/G0 phases that were accompanied by increased cell MSC numbers in G2/M phases following RT was slightly reversed to the numbers estimated for control conditions (**Figure 2B**). It is worth noting that cell death induction, particularly following a combined RT treatment, could not be observed (**Figure 2B**, subG1 fractions). Thus, MSCs are quite resistant with apoptosis rates <15% (96 h post-treatment). The kinetics of DNA double-strand break repair, as analyzed by phosphorylation via immunofluorescence, revealed that NSCLC-conditioned media treatment tended to reduce the RT-induced DNA damage response (**Supplementary Figure S2**). NSCLC-conditioned media-treated MSCs showed generally reduced numbers of H2A.X foci, an effect that was significant at 2 h post-treatment, indicating an improved repair capacity following stimulation with NSCLC-derived factors. Treatment with H460-conditioned media further reduced the colony formation capacity of MSCs, while treatment with A549-conditioned media showed no alterations (**Figure 2C**). Both treatments seemed to induce resistance in MSCs as revealed by improved clonogenic survival rates, especially when the treatment with H460-derived conditioned media was combined with RT (**Figures 2D, E**). Besides the colony formation capacities of the MSCs upon treatment, the differentiation potential was investigated as an additional important stem cell feature. While the trilineage potential was not affected following NSCLC cell-conditioned media treatment (not shown), it was found to be reduced following RT treatment (**Supplementary Figure S3A**). Interestingly, we noted that MSCs showed an altered morphology, particularly following RT with enlarged, more flattened, and irregular cell shapes, as typically seen in radiation-induced senescence (**Figure 2F**). RT-induced senescence formation was specified in MSCs following NSCLC-conditioned media treatment using flow cytometry in combination with the fluorescent dye C12FDG, which can be hydrolyzed by β galactosidase enriched in lysosomes upon senescence yielding in green fluorescence (**Figure 2G**, **Supplementary Figure S3B**). According to the reduced proliferation rates stated above following tumor-conditioned media treatment, an increase in senescence was already detectable under non-irradiated conditions (**Figure 2G**). RT clearly induced senescence in MSCs, independent of concurrently applied conditioned media treatments. The protein expressions of the senescence markers cyclin-dependent kinase inhibitor CDKN1A/p21/Waf1/Cip1 and cyclin D1 (CCND1) were further determined by Western blot analyses, and the expression levels were clearly induced upon irradiation, while the expression levels of the proliferating cell nuclear antigen (PCNA) were reduced, without obvious effects concerning the NSCLC-conditioned media treatments (**Figure 2F**).

Thus, NSCLC-derived factors had “some” effect on MSCs, with noted significant reductions in proliferation potential that were associated with increased resistance traits after combined RT treatment.

### 3.2 The phenotypic changes of MSCs by NSCLC-derived factors and, particularly, RT-induced senescence were confirmed using a more direct co-culture approach using Transwell co-cultures

**Figure 3:**
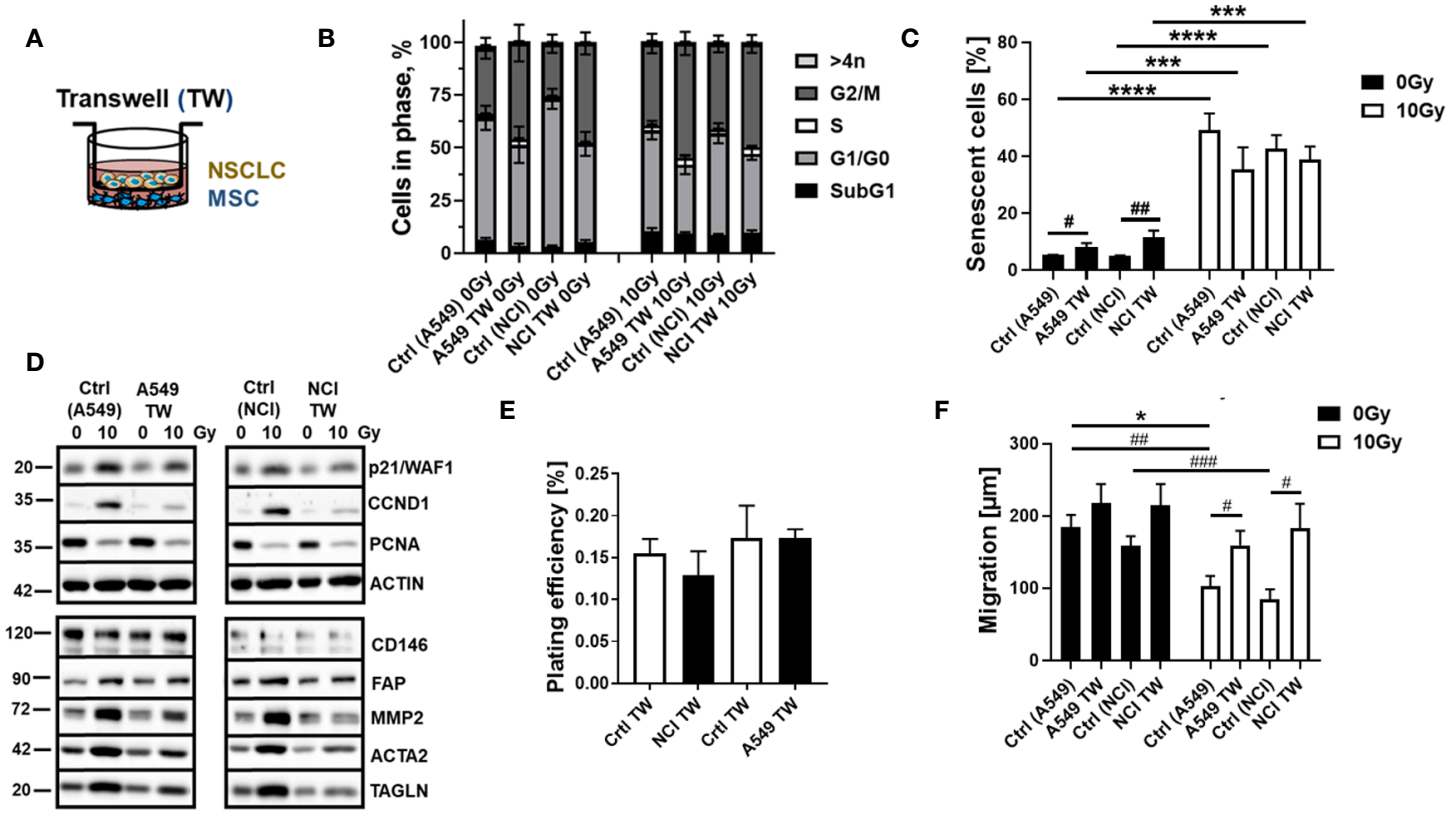
RT-induced senescence of MSCs was not affected by the combined treatment with NSCLC-derived factors in a more direct co-culture approach using Transwell co-cultures. MSCs were cultured alone (“empty” Transwell) or together with NCI-H460 and A549 (in indirect co-culture) for 24 h prior to RT with 0 or 10 Gy and analyzed after additional 96 h **(A)**. Cycle phases and apoptotic cells (subG1) of MSCs were analyzed by flow cytometry **(B)**. The graphs consist of data from four to eight individual experiments (with SEM). The statistically significant differences within G1/G0 [Ctrl (NCI) 0 Gy vs. NCI TW 0 Gy: *p* ≤ 0.01; Ctrl (NCI) 0 Gy vs. Ctrl (NCI) 10 Gy: *p* ≤ 0.001], and G2/M phases [Ctrl (NCI) 0 Gy vs. NCI TW 0 Gy: *p* ≤ 0.01; Ctrl (NCI) 0 Gy vs. Ctrl (NCI) 10 Gy: *p* ≤ 0.05] as estimated by two-way ANOVA with Tukey’s multiple-comparison test were not depicted. **(C)** The senescence induction of the respective MSC cultures were analyzed by C12FDG treatment prior to flow cytometry analyses 96 h post-treatment. The graphs depict data from four to six independent experiments. *P*-value by two-way ANOVA, followed by *post hoc* Tukey’s multiple-comparison test: ***P ≤ 0.005 ****P ≤ 0.001 and additionally by unpaired (two-tailed) *t*-tests depicted as ^#^ *p* ≤ 0.05; ^##^ *p* ≤ 0.01. **(D)** The expression levels of the indicated proteins were analyzed in whole protein lysates of cultured MSCs with or without radiation treatment (96 h after RT with 10 Gy) in the presence co-cultured NSCLC cells using Western blot analysis. Representative blots from at least three independent experiments are shown. **(E)** The colony formation capacity was further evaluated by plating MSCs at low densities (100–250 cells/well) in plastic culture dishes and subsequent culturing 10 days in the presence or absence of co-cultured NSCLC. Coomassie Brilliant Blue-stained MSC colonies were counted, and the plating efficiency was calculated. The graphs depict data from three to four independent experiments, each measured in duplicates. **(F)** MSC migrations were investigated 96 h after irradiation with 10 Gy following the introduction of a thin wound in confluent monolayers by scratching with a pipette tip. Wound closure was determined following NSCLC co-culture by measuring the migration distance (wound closure) after 12 h. Wound closure was related to the distance of the introduced wound, and the migrated distance was calculated. Data are shown as means ± SEM of three to five independent experiments. *P*-value by two-way ANOVA, followed by *post hoc* Tukey’s multiple-comparison test: *P ≤ 0.05 and additionally by unpaired (two-tailed) *t*-tests depicted as ^#^ *p* ≤ 0.05, ^##^ *p* ≤ 0.01, and ^###^ *p* ≤ 0.005.

The concurrent application of RT seemed to cause crucial changes in the cellular characteristics of MSCs more than the tumor-related factors. RT particularly induced a senescence phenotype of MSCs. To ensure the previous results and further investigate how “chemical signals” released by the NSCLC cells impact on MSCs while minimizing the concentration, processing, and storage artifacts of the tumor-secreted factors, we repeated the experiments in a more direct approach using Transwell co-culture systems (**Figure 3**). Concerning the cell cycle distributions, the co-culture of MSCs with NCI cells resulted in reduced MSC numbers in G1/G0 phases while increasing MSCs in G2 phases, effects that were only seen by tendency for MSCs within the respective A549 co-cultures (**Figures 3A, B**). Combined RT treatments even did not obviously affected the MSC cell cycle distributions in A549 co-cultures (except for a slight G2/M arrest), while a clear RT-induced G2/M arrest was less pronounced in MSCs when co-cultured with NCI cells. RT-induced cell death levels as indicated by low (<10%) subG1 fraction values again showed the quite resistant phenotype of MSCs at 96 h post-treatment (**Figure 3B**). RT-induced senescence induction was further confirmed following RT with no further alterations due to factors derived from co-cultured NSCLC cells (**Figure 3C**). Western blot analyses of the CDKN1A/p21 and CCND1 protein levels showed again increased expression levels following RT, while PCNA levels were declined (**Figure 3D**). Concerning potential phenotypical alterations, RT seemed to increase the expression levels of FAP (fibroblast activation protein), MMP2 (matrix metalloproteinase 2), and ACTA2 (alpha smooth muscle actin) as well as TAGLN (transgelin), indicating an activated, more reactive MSC phenotype or potential differentiation toward a cancer-associated fibroblast/myofibroblast-like phenotype (**Figure 3D**), although induced expressions were not significant, and no significant alterations concerning classical MSC marker expressions could be found (not shown). This might indicate that MSCs within lung cancer stay as phenotypically similar MSCs but with altered cancer-promoting features, having become tumor-associated MSCs (TA-MSCs). Among the cellular features, the colony formation capacity was not affected upon NSCLC co-culture (**Figure 3E**), while the migration of MSCs tended to be increased in these cultures, an increase that became significant following co-applied RT, although RT itself caused a reduction in respective migration capabilities (**Figure 3F**).

Conclusively, in direct NSCLC–MSC co-cultures, MSCs were able to mediate NSCLC cell resistance to RT. Although NSCLC cells or particularly NSCLC cell-derived factors impacted on MSCs’ cellular features, co-applied RT acted as a crucial element for the phenotypic MSC changes, finally resulting in MSC senescence.

### 3.3 The senescent phenotype of RT-treated MSCs is characterized by increased expression and secretion of SERPINE1, a potential RT mediator

**Figure 4:**
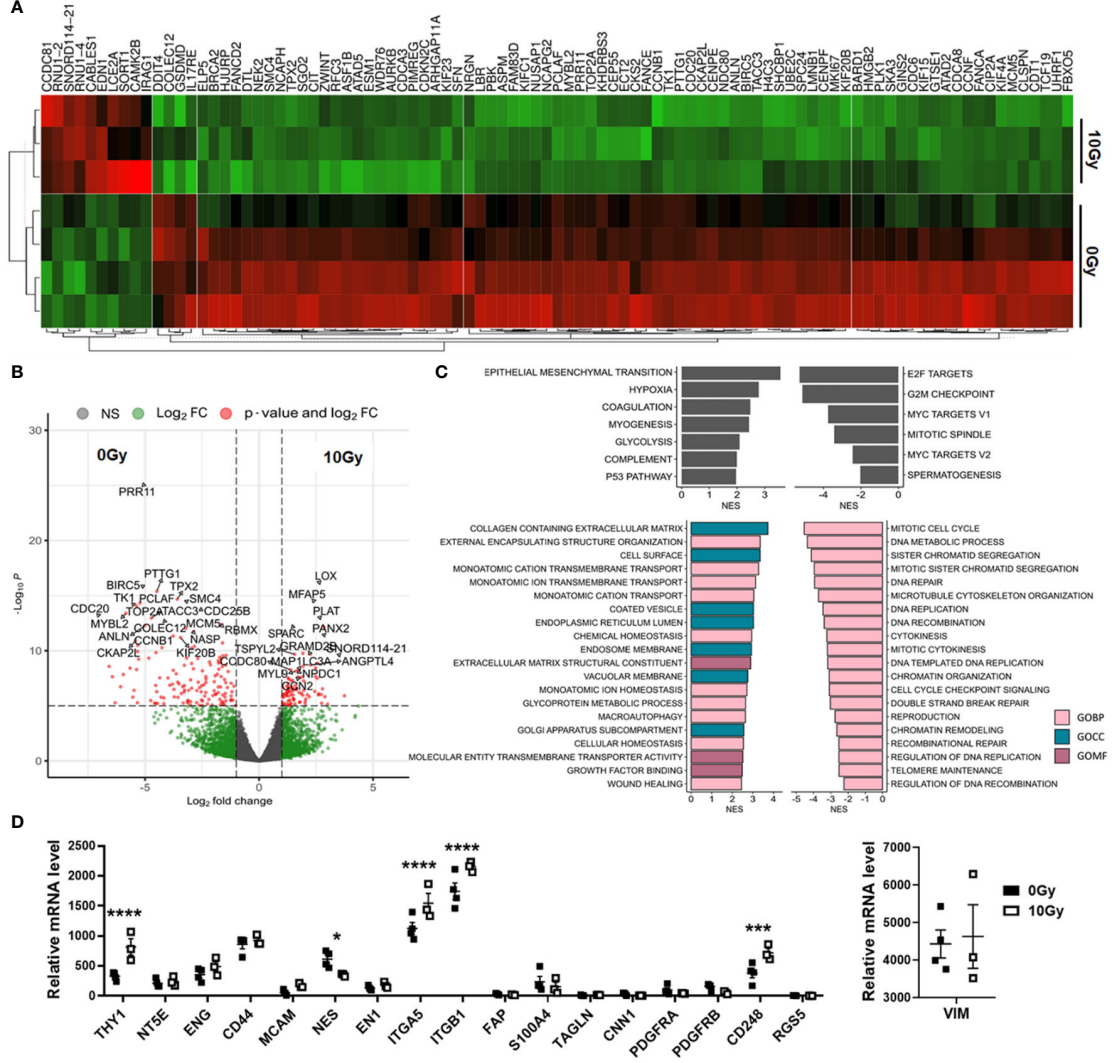
Global gene expression analysis revealed differences in MSCs upon RT. Total RNA (whole transcriptome) sequencing was performed using total RNA isolates of cultured MSCs following RT with 0 Gy (Ctrl) and 10 Gy at 96 h post-treatment. **(A)** Hierarchical clustering heatmap of genes with log-fold change >0.5 and *p*-value <0.001 (top 100 variant genes) and **(B)** Volcano plot with all genes (13,636 variables in total) are shown. Significant differences in 1,049 transcripts (fold change >1.5 and adjusted *p*-value cutoff of 0.001) were highlighted in red. Genes upregulated in Ctrl MSCs are on the left, while genes upregulated in following RT are on the right site. The top 25 genes are named. **(C)** Significantly enriched Hallmark and significantly enriched C5 ontology gene sets showing normalized enrichment scores (NES) for significantly upregulated and downregulated gene sets during RT are shown. BP, biological process; CC, cellular component; MF, molecular function. **(D)** Relative mRNA expression levels obtained from the RNAseq profiles of classical MSC signature genes are separately depicted. Biological replicates as indicated: MSC 0 Gy *n* = 4; MSC 10 Gy *n* = 3. Statistics: limmas moderated *t*-test, adjusted by “BH” with *p ≤ 0.05, ***p ≤ 0.005, ****p ≤ 0.001.

**Figure 5:**
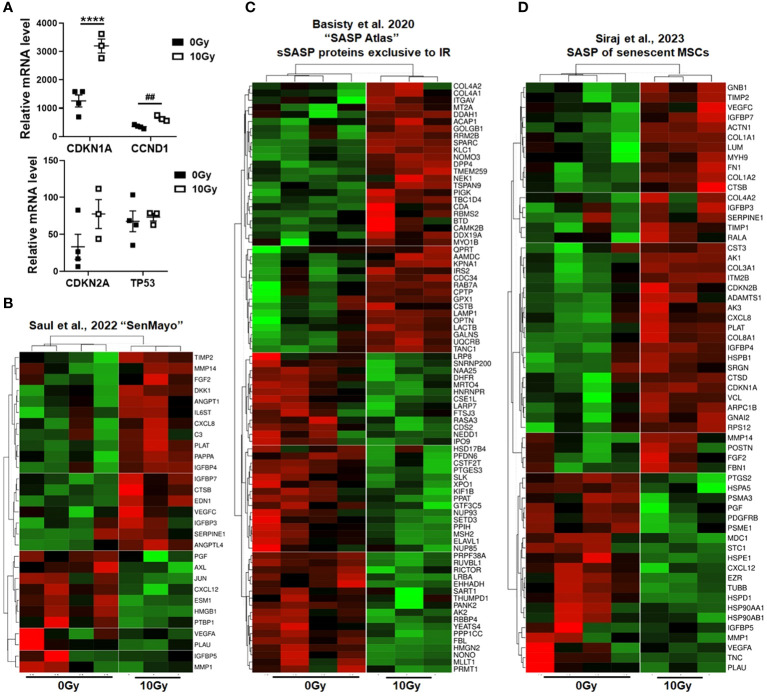
Senescent phenotype of RT-treated MSCs. **(A)** Relative mRNA expression levels as obtained from the RNAseq profiles of the indicated senescence marker genes are depicted. Data are shown as means ± SEM of the indicated biological replicates (MSC 0 Gy *n* = 4; MSC 10 Gy *n* = 3). *P*-value by one-way ANOVA, followed by *post hoc* Tukey’s multiple-comparison test: ****P ≤ 0.001 and additionally by unpaired (two-tailed) *t*-tests depicted as ^##^ *p* ≤ 0.01. **(B–D)** Gene set enrichment analysis (GSEA) using specific senescence gene sets are shown: **(B)** the ‘SenMayo’ gene set identifying senescent cells across tissues (34), **(C)** the ‘SASP Atlas’ comprising soluble proteins and exosomal cargo SASP factors exclusive to ionizing radiation (35), and **(D)** the SASP gene set of senescent MSCs (36). Displayed are heatmaps of significantly enriched genes.

In order to prove the senescent phenotype of irradiated MSCs and to get an idea about the involved signaling pathways and moreover which candidate factors might be involved in NSCLC progression and RT resistance, we performed gene expression profiling by RNA sequencing (RNA-Seq), comparing non-irradiated MSCs with senescent MSCs at 96 h postRT (**Figure 4**). A hierarchical clustering was built based on the expression levels of the top 100 variant genes (with log-fold change >1.5 and *p*-value <0.001), identifying signatures that validated at the transcriptome level the RT-induced changes following RT (**Figure 4A**). The volcano plot analysis further highlighted differential gene expressions in “healthy” and senescent MSCs (**Figure 4B**). Using the log-fold change >1.5 and the adjusted *p*-value of <0.001, 1,049 transcripts were found to be differentially expressed in both cell states, with 405 genes found to be upregulated in senescent MSCs compared to 644 upregulated genes in control (non-irradiated) MSCs (**Figure 4B**). Gene set enrichment analysis (GSEA) was performed according to the Hallmark gene set (including 50 gene sets of specific, well-defined biological states or processes) and the C5 ontology gene sets (including 15,937 gene sets containing biological process, cellular component, and molecular function components) (**Figure 4C**) from the molecular signature database MSigDBv6.1 (Broad Institute). In “healthy” non-irradiated MSCs, GSEA revealed a strong signature for, e.g., epithelial–mesenchymal transition (EMT), which is known to be regulated by MSCs even in tumor cells following crosstalk between tumor cells and MSCs, regulation of coagulation and complement being decisive for resolving inflammatory processes, particularly important for the regenerative capacity of healthy MSCs together with wound healing, and the p53 pathway playing a critical role in cellular responses to stress as well as genes involved in vesicular and transmembrane transport activities indicating exocytosis features. Gene sets upregulated in irradiated and thus senescent MSCs addressed predominantly critical regulators of genes required for cell cycle progression, and that play an integral role in the control of cell proliferation. In contrast, classical mesodermal and MSC-related marker genes, including CD73/NT5E, CD105/ENG, CD146/MCAM, VIM (vimentin) and ENG1 (engrailed 1), were not affected at 96 h following RT treatment, still indicating the “MSC” phenotype (**Figure 4D**). Significant increases were found in mRNA expression levels for CD90/THY1, the integrins ITGA5 and ITGB1, together with CD248 (also known as endosialin, a transmembrane receptor whose known ligands are the extracellular matrix components fibronectin and type I/IV collagen), which fits with the activated phenotype and increased migratory capabilities of treated MSCs. A potential switch in phenotypic markers, e.g., FAP, S100A4, TAGLN, CNN1, and RGS5, that should be affected upon induced differentiation into fibroblast, pericytes, and/or smooth muscle cells, could not be observed. The senescence marker genes CDKN1A/p21 and CCND1 were clearly induced following RT on mRNA level together with the INK4 family member p16 and p53 only by tendency (**Figure 5A**). To further confirm that RT-treated MSCs displayed a senescent gene expression signature corresponding to well-known senescent (MSC) phenotypes reported by others, we performed GSEA again (**Figures 5B-D**). Using the “SenMayo” gene set allowing the identification of senescent cells and prediction of senescence-associated pathways across tissues (34), the senescence phenotype of irradiated MSCs was confirmed, and further revealed relevant SASP candidates: angiogenic growth factors (e.g., ANGPT1, EDN1, FGF2, VEGFC), coagulation-and complement-related, and thus inflammation-related factors (e.g., C3, PLAT, SERPINE1, CXCL6, IL6st), as well as factors involved in ECM remodeling (e.g., MMP14, TIMP2) (**Figure 5B**). Similar observations could be seen for secretory SASP factors exclusive to ionizing radiation as previously described (35) (**Figure 5C**) and the composition of SASP released by senescent MSCs (36) (**Figure 5D**).

**Figure 6:**
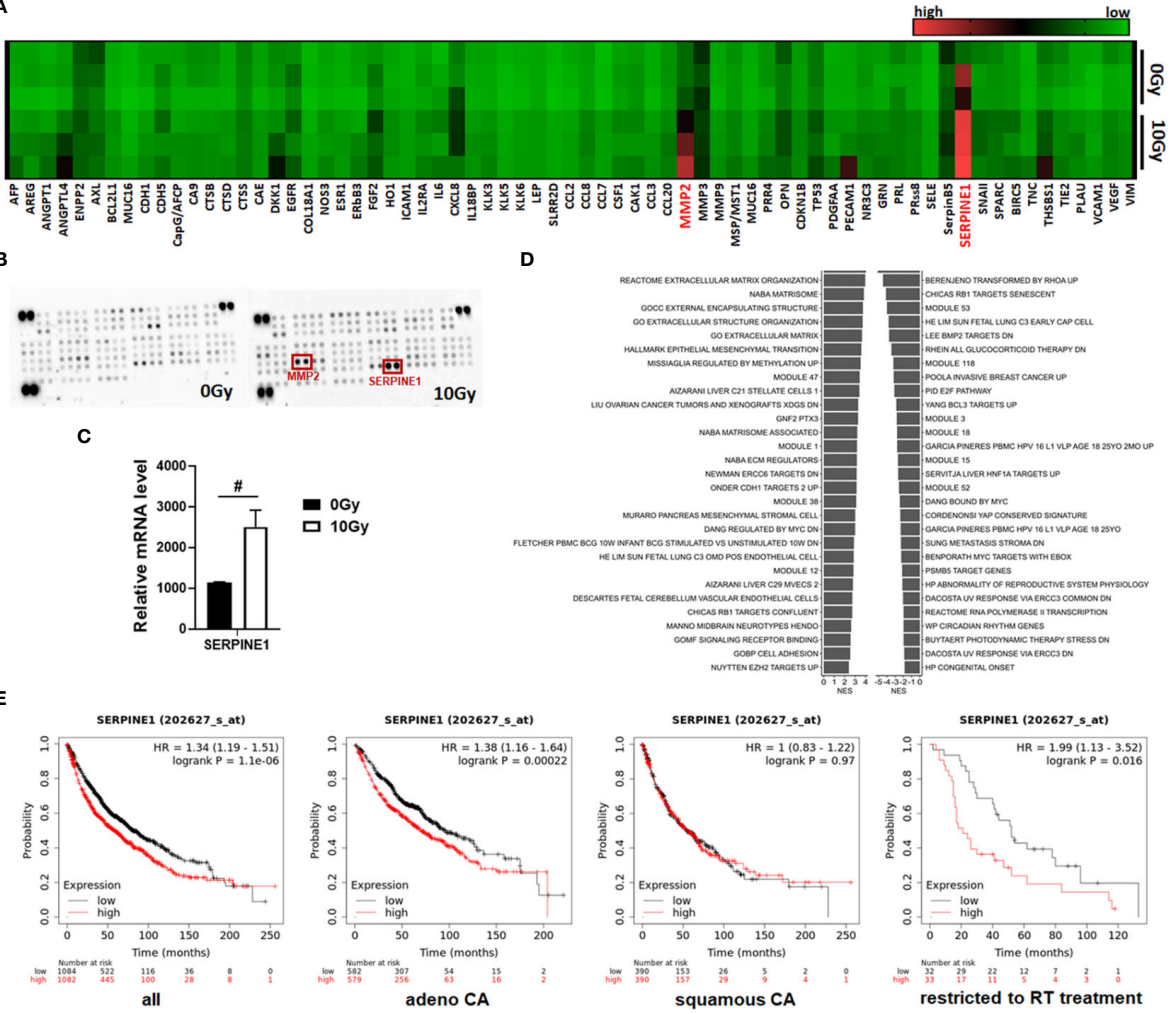
MSCs that have become senescent during RT express and secrete SERPINE1, a potential RT mediator. **(A, B)** A membrane-based sandwich immunoassay (Proteome Profiler Human XL Oncology Array, R&D Systems) was used for the parallel determination of the relative levels of selected human cancer-related proteins and cytokines in cell culture supernatants of control (0 Gy) and RT-treated (10 Gy) MSCs. **(A)** Profiles of detected signals were quantified by densitometry, related to the reference signal, and are presented as heatmap. Biological replicates are indicated: MSC 0 Gy *n* = 3; MSC 10 Gy *n* = 3. **(B)** Representative membranes of the 0 Gy and 10 Gy conditions are exemplarily shown. Select analytes (matrix metalloproteinase 2, MMP2, and serine proteinase inhibitor E1, SERPINE1) being secreted in increased amounts following RT-induced senescence in MSCs are highlighted in red. **(C)** Relative mRNA expression levels obtained from the RNA-Seq profiles of SERPINE1 (also known as plasminogen activator inhibitor-1, PAI-1) are depicted. Data are shown as means ± SEM (biological replicates: MSC 0 Gy *n* = 4; MSC 10 Gy *n* = 3). *P*-value by unpaired (two-tailed) *t*-test depicted as ^#^ *p* ≤ 0.05. **(D)** Significantly enriched gene sets comprising the SERPINE E1 gene and showing normalized enrichment score (NES) for significantly upregulated (right site) and downregulated (left side) gene sets following RT in MSCs are shown. **(E)** Survival curves concerning SERPINE1 gene expressions were plotted for all NSCLC patients (*n* = 2,166), adenocarcinoma (adeno CA) patients (*n* = 1,161), and squamous cell carcinoma (squamous CA) patients (*n* = 780) as well as for all available patients treated with RT (*n* = 65). Data was analyzed using Kaplan–Meier plotter for lung cancer (www.kmplot.com). Expressions in cancer tissues above the median are indicated in red line, and expressions below the median are summarized in black line. Number-at-risk values are shown below the main plot, and log rank *p*-value as well as hazard ratio (HR) with 95% confidence intervals are indicated.

As expected, these results revealed numerous soluble SASP factors, potentially secreted irradiated MSCs that, in turn, could impact on the NSCLC response to cancer therapy, particularly to RT, and could—at least partially—induce resistance. To further confirm our findings and to gain more insight how senescent MSCs could mediate these actions, we further performed multiplex antibody arrays to detect cytokines, chemokines, and acute-phase proteins in conditioned medium harvested from MSCs either under non-irradiated or irradiated conditions (**Figures 6A, B**). It is worth noting that the array membranes incubated with the conditioned media from irradiated MSCs featured a predominant hybridization signal for the matrix metalloproteinase MMP2 and the serine protease inhibitor (serpin) SERPINE1, also known as plasminogen activator inhibitor-1 (PAI-1). Increased SERPINE1 expression levels in senescent MSCs were confirmed in the RNA-Seq datasets (**Figure 6C**). For MMP2, known to play an important role in the process of stem cell migration as well as for tissue remodeling favoring tumor progression and cell extravasation, only an increase in mRNA expression levels by tendency could be revealed, together with a general increase of the MMP2–MMP14–TIMP2 axis and associated ECM genes (**Supplementary Figure S4**). GSEA was again performed using all gene sets that include SERPINE1 (**Figure 6D**). In particular, gene sets that contain genes associated with cancer were found to be significantly changed in the irradiated MSCs, e.g., certain cancer modules and metastasis or invasion gene sets, together with (already identified) gene sets addressing senescence or cell cycle regulations. We then studied the relationship between mRNA expressions of SERPINE1 and the clinical outcome using a Kaplan–Meier plotter for lung cancer (**Figure 6E**). Survival curves were plotted for all NSCLC patients and separately for adeno and squamous NSCLC patients. SERPINE1 high mRNA expression was found to be correlated to worse overall survival (OS) for all NSCLC patients, particularly when considering adenocarcinoma patients only. SERPINE1 expression in squamous carcinoma did not significantly affect OS. Above all, it was shown that, in the subgroup of all NSCLC patients who received radiotherapy (and this concerned 65 patients exclusively with adenocarcinoma), an increased expression of SERPINE1 correlated with a significantly worse overall survival (**Figure 6E**). Considering other SASP factors secreted by irradiated MSCs, particularly MMP2, high mRNA expression levels did not correlate to worse OS for all NSCLC patients, but when considering adenocarcinoma patients only (**Supplementary Figure S4**). An additional effect in the subgroup RT-treated NSCLC patients could not be determined.

Thus, lung-resident MSCs, which are already known to be key players in the development and malignancy of lung cancer, can be negatively influenced, namely become senescent, by applied RT. The associated increased SASP factor expression and, particularly through SERPINE1, have the potential to mediate RT resistance in NSCLC.

## 4 Discussion

The hitherto normal tissue-resident MSCs, which are known to be important tissue orchestrators in healthy lung homeostasis and pulmonary tissue repair, can be affected upon cancer carcinogenesis, namely, recruited and activated to acquire tumor-promoting properties (12, 37). Cancer cells, so-called "seed" herein, modulate their surrounding stroma (including locally residing MSCs) as “soil”, finally causing an “activated” tumor-promoting microenvironment (22, 38, 39). At the same time, MSCs seem to exhibit an inherent tropism toward primary tumors and metastatic sites besides their ability to migrate to inflammatory and fibrotic sites (10, 23, 40, 41). Numerous *in vitro* and *in vivo* investigations already highlighted that mesodermal cells foster NSCLC progression, e.g., by increasing lung cancer cell proliferation, survival, and invasiveness via inducing EMT as well as by mediating the expansion of immunosuppressive immune cell subsets (40, 42–46). However, a definitive cell fate of “educated” MSCs remains elusive. MSCs could potentially represent precursor cells for other mesodermal stromal cells, particularly activated, cancer-associated fibroblasts, contributing to the heterogeneity and complexity of lung cancer or for vascular mural cells fostering vascular remodeling (12). MSC differentiation towards these cell types could not be convincingly shown in the present study as no obvious alterations of phenotypic markers could be observed following the co-culture with NSCLC cells. This might be due to the fact that unique MSC phenotypic markers similar to other mesodermal markers are missing.

**Figure 7:**
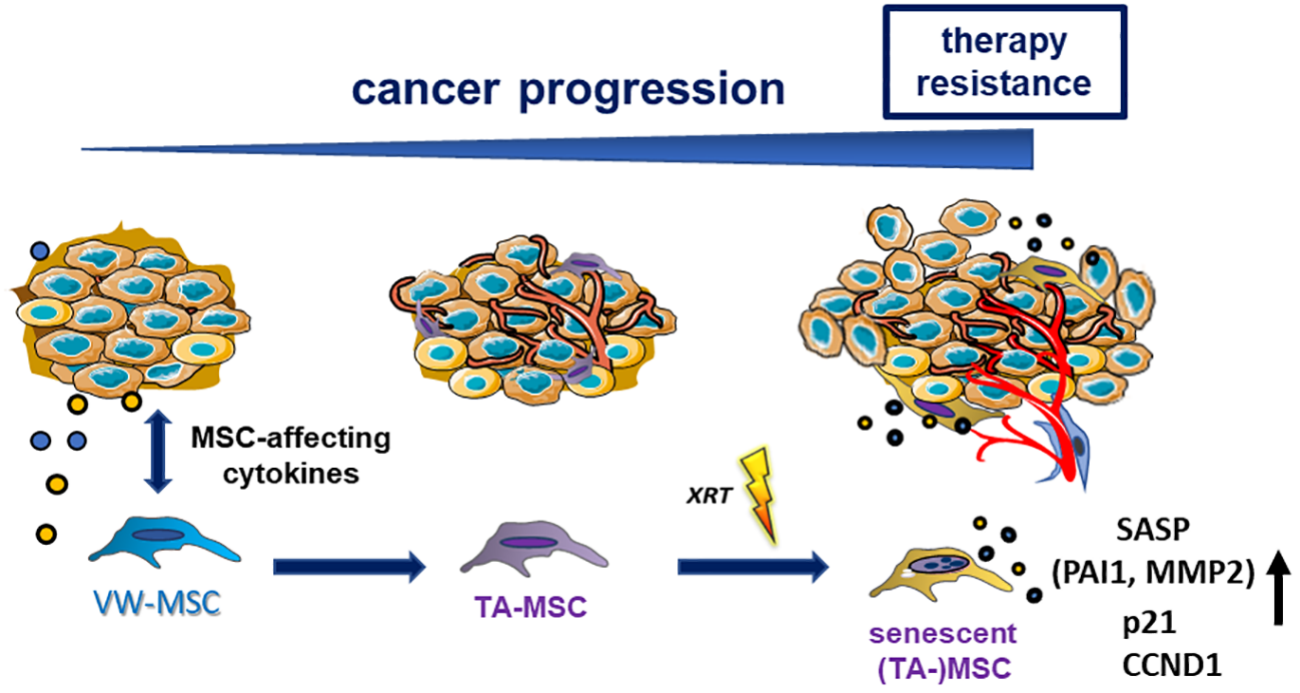
Summarizing scheme. Local (tissue-resident) MSCs could be activated, recruited, and then “educated” by tumor cells and particularly by tumor-secreted (and MSC-affecting) factors. MSCs, in turn, adopt altered cellular features, e.g., a tumor-promoting secretory profile, thus becoming tumor-associated MSCs (TA-MSCs). Phenotypical alterations can further be induced by applied cancer therapeutics (e.g., radiation therapy-induced senescence), finally re-enforcing cancer progression and therapy resistance. Senescent TA-MSCs express and secrete PAI1/SERPINE1, a potential RT mediator.

Based on their definition as multipotent stromal cells, which can be identified by plastic adherence, expression of CD90, CD73, CD105, and CD44 while lacking hematopoietic markers, and the differentiation potential toward mesodermal lineage cells (e.g., adipocytes, chondrocytes, and osteocytes) (47, 48), it is not possible to claim a “unique” MSC phenotype while excluding other mesodermal cell types (32, 49). Thus, we speculate that MSCs stay as MSCs with tumor-promoting cellular features, e.g., a tumor-supporting secretory profile, thus having become tumor-associated MSCs, an observation that is particularly fostered by co-applied cancer therapeutics, namely, RT finally re-enforcing cancer progression and therapy resistance as investigated here (**Figure 7**). Radiotherapy treatment is capable of inducing senescence in MSCs and the accompanying hypersecretion phenotype, defined as the senescence-associated secretome, includes the SASP factor PAI1/SERPINE1, a potential RT mediator, which, in turn, affects the (RT-) phenotype of NSCLC cells. Compared to the rather low numbers of MSCs within healthy lungs, an increased numbers were found in NSCLC tissues (10, 14, 50). The reported upregulation of MSC-specific identity genes, namely, certain HOX genes in these NSCLC tissues and particularly in the MSCs here, already indicated that recruited and somehow educated MSCs are still MSCs but became tumor-associated MSCs contributing to important and decisive (stromal) features characterizing the histologic subtypes (14). For NSCLC, similar to other desmoplastic cancers (e.g., breast and pancreatic cancers), it was already suggested that the cancer cells here together with co-applied cancer therapy educate recruited MSCs to acquire a tumor-associated MSC phenotype that was characterized by the secretion of several pro-stemness growth factors, chemokines, and interleukins, finally shaping the pro-tumorigenic microenvironment and thus contributing to the definition of the histological types in NSCLC (51, 52). Tumor-associated MSCs furthermore exhibit stromal biomarker potential that could be addressed to improve risk stratification and prognosis prediction for lung cancer patients and could even represent an additional therapeutic target to manipulate the interaction between MSCs and NSCLC cells (12, 14, 40, 53). Especially following RT, senescent MSC-secreted bioactive SASP factors could serve as biomarker candidates.

Nowadays, it is clear that the therapeutic efficacy of RT is not limited to the capacity to directly kill malignant cells. Even the non-malignant stromal cells within a tumor receive the same prescribed radiation dose during treatment, and RT-induced senescence of tumor stromal cells turned out to be decisive for treatment failure and tumor progression (54). The accumulation of senescence-like fibroblasts particularly was already demonstrated to be the primary common pathologic mechanism of immunotherapy- and irradiation-induced lung injury (55). The omnipresence of MSCs throughout vascularized organs and thus within the lungs (10, 12, 14, 56–58) strongly suggests that these cells could serve as “first line” cells ready to be rearranged with the tumor stroma and thus are subjected to cellular senescence by genotoxic stressors that, in turn, increase the complexity in (cancer) diseases and accompanied immune system regulations by the autocrine and paracrine properties of senescent MSCs (59). We used the “SenMayo” gene set to identifiy senescence and senescence-associated pathways (34) as well as the secretory SASP protein panel (the “SASP atlas”) exclusive to IR (35) and the SASP panel released by senescent MSCs (36) (i) to confirm the senescent phenotype of irradiated MSC and (ii) to reveal relevant SASP factors that could meditate NSCLC RT resistance. Among the SASP factors, serine protease inhibitors and particularly SERPINE1 (serpin family E member 1), as confirmed by proteome profiling, were revealed as relevant MSC-derived candidate SASP factors potentially mediating RT resistance. The respective Kaplan–Meier results confirmed that NSCLC patients with a high SERPINE1 expression exhibited worse overall survival, particularly those with lung adenocarcinoma and following RT.

Within its function as an important regulator of extracellular matrix remodeling, increased SERPINE1 expression levels were already associated with poor prognosis in cancer (60–63). Although not derived from lung cancer studies, a role of SERPINE1 for the remodeling of the tumor microenvironment of the colon, including infiltration of immune cells, was reported (62). Confirming studies described the potential of SERPINE1 to promote neovascularization in gastric tumors and thus interacting with inflammation (61). Moreover, using indirect co-cultures of human (bone marrow-derived) MSCs together with (esophageal squamous) carcinoma cells, it was revealed that upon co-culture, MSCs adopted a more CAF-like phenotype accompanied by increased SERPINE1 expression levels that, in turn, induced migration and invasion abilities in the cancer cells (64). SERPINE1 enrichment within the tumor microenvironment was also observed in (triple-negative) breast cancer, and inhibition strategies markedly limited the aggressive phenotype of respective breast cancer cells, even reduced radioresistance (65). Herein the role of increased SERPINE1 levels was linked to an improved DNA damage response in an ATM/ATR-dependent manner, finally mediating resistance to RT (65). Concerning lung cancer, SERPINE1 expression turned out to be upregulated (in a TGFβ−mediated and Yes−associated protein−dependent manner) in mesenchymal lung cancer cells that promoted their invasiveness (60). SERPINE1 (together with SERPINB7) was further described as an unfavorable prognostic prediction of overall survival in NSCLC patients receiving the standard of care (66). We identified SERPINE1 here as a crucial MSC-derived SASP factor with the potential to mediate NSCLC radioresistance. SERPINE1 (together with ANGPTL4, GDF15, CXCL12, SOX9, HOXB6, and ANK3) was even described as a TA-MSC factor, namely, in mesenchymal stromal cells of the bone marrow that supported myeloma cell growth (67). Importantly, SERPINE1 expression was linked here to a senescent MSC phenotype characterized by an increased expression of senescence-associated β-galactosidase, increased cell size, and reduced proliferation capacity (67). Serine protease inhibitor expression, was further shown to mediate host immune response evasion (68) due to the regulatory interactions of peptidase inhibitors of the SERPIN superfamily with granzyme B activities of lymphocytes (69). Thus, increased SERPINE1 expression and secretion levels of senescent MSCs within the tumor stroma could contribute to the ability of the “system” to escape immune cell-mediated lysis. Generally, the active cellular phenotypes of RT-induced senescent stromal cells, particularly of MSCs, affect themselves and neighboring cells via autocrine and paracrine patterns, including the promotion of tumor progression, distant metastasis, immune infiltration, and therapy resistance (70). SASP factors released by senescent stromal cells were shown to cause TGF-dependent EMT, increasing invasiveness, non-cancer stem cell (CSC) dedifferentiation toward more malignant CSCs, and remodeling of the tumor extracellular matrix, as well as to mediate tumor immunity (e.g., inflammation promotion immunosuppressive cell recruitment) (70). Within that scenario, specifically induced apoptosis of senescent tumor stromal cells following RT resulted in remarkable effects on radiosensitizing NSCLC cells *in vitro* and *in vivo* and even improved radiation-induced pulmonary fibrosis (71). Due to the critical role of RT in both curative and palliative treatments of NSCLC, additional radiosensitizers, and/or alternative therapeutic targets, e.g., “factors” of the tumor microenvironment are urgently needed to improve the tumor’s response to RT as radioresistance could not be overcome by “simply” increasing the RT doses applied because of the maximum RT dose limitation by normal tissue tolerance. These observations, together with the results presented here, strongly suggest that stromal and, more precisely, senescent TA-MSCs derived SERPINE1/PAI1 contributes to the progression and resistance of NSCLC, making it a potential target for lung cancer therapy.

## Data availability statement

The datasets presented in this study can be found in online repositories. The names of the repository/repositories and accession number(s) can be found in the article/**Supplementary Material**.

## Ethics statement

The studies involving human material were reviewed and approved by the local ethics committee (Ethik-Kommission, Universität Duisburg-Essen, Medizinische Fakultät, Essen)University, Medical Faculty, Essen, Germany (nos. 104363 and 177454-BO). The participants provided their written informed consent to participate in this study.

## Author contributions

HS: Data curation, Formal analysis, Validation, Visualization, Writing – original draft, Writing – review & editing. AB: Data curation, Validation, Writing – original draft, Writing – review & editing. BB: Data curation, Formal analysis, Methodology, Validation, Visualization, Writing – original draft, Writing – review & editing. DK: Conceptualization, Data curation, Formal analysis, Funding acquisition, Investigation, Methodology, Project administration, Resources, Supervision, Validation, Visualization, Writing – original draft, Writing – review & editing.
