# Supplementary material for: Non-small cell lung cancer cells and concomitant cancer therapy induce a resistance-promoting phenotype of tumor-associated mesenchymal stem cells
Source: Front Oncol. 2024 Jul 1;14:1406268. doi: 10.3389/fonc.2024.1406268 (PMC11246879; doi:10.3389/fonc.2024.1406268)
Supplement: Supplementary file 1 [file Presentation_1.pdf]

Supplementary Material

1 Supplementary Figures

Supplementary Figure S1

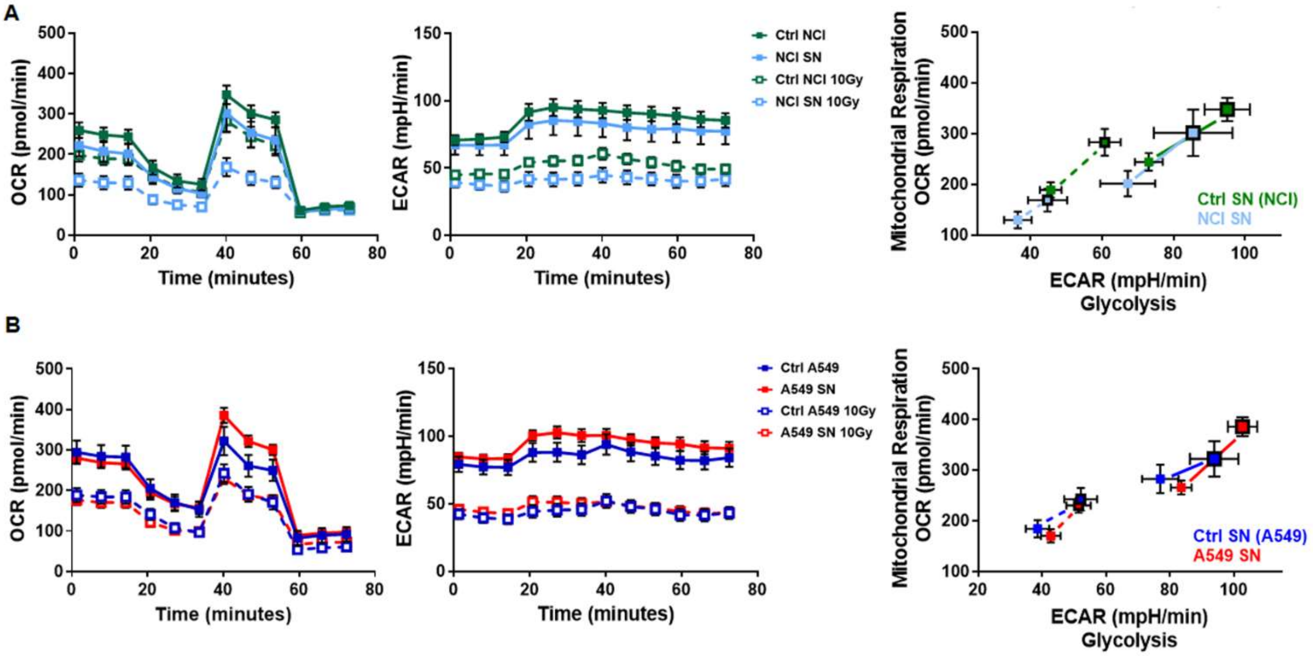

Supplementary Figure S1: Treatment of cultured MSCs with tumor secreted factors derived from NSCLC cells did not obviously affect the metabolic potential, while respective reductions were observed following RT. The metabolic potential of cultured MSCs was analyzed after treatment with tumor conditioned media (SN; derived from cultured NCI-460 and A549 NSCLC cells) in combination with or without radiation treatment (RT; 10Gy) after 48 hours. Therefore, oxygen consumption rates (OCR) and extracellular acidification rates (ECAR) were measured in MSCs over time, while oligomycin (Oligo; 1 µM), FCCP (1 µM) and rotenone/ Antimycin A (Rot/Ant; 0.5 µM) were added at indicated time points. OCR levels and ECAR measurements over time are shown for combined (**A**) NCI-H460 SN- and (**B**) A549 SN-RT treatments. Data were summarized as mean values ± SD (measured in 4–7 replicates each). The relative utilization of the two energy pathways mitochondrial respiration and glycolysis, as estimated by OCR and ECAR over time were summarized upon both baseline and stressed conditions following RT treatment (0Gy: solid line; 10Gy: dashed line) (**A**, **B**, right panels).

Supplementary Figure S2

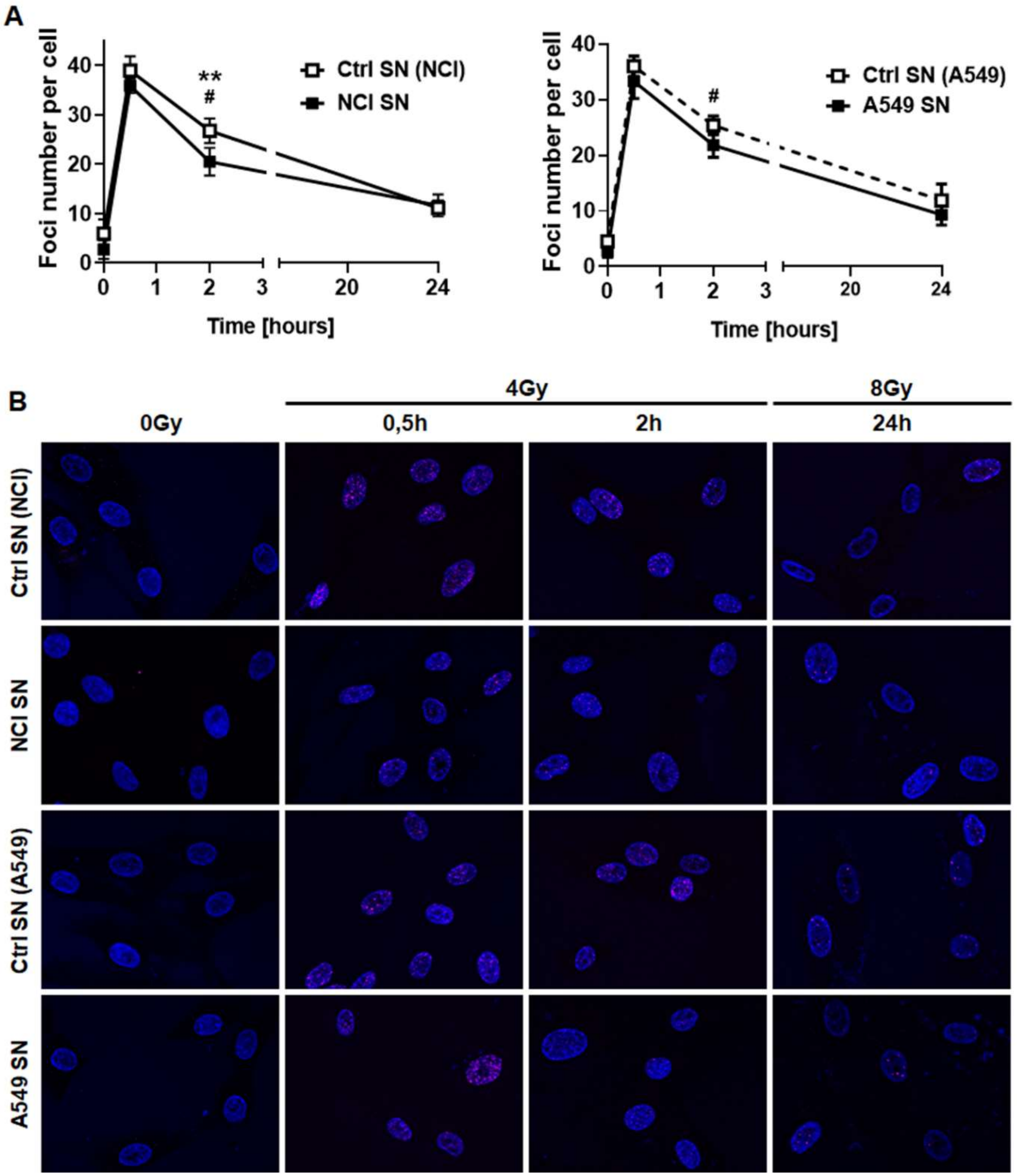

Supplementary Figure S2: DNA damage repair kinetics of cultured MSCs treated with tumor secreted factors derived from NSCLC cells following RT. RT-induced DNA double-strand breaks (DSBs) as one of the most critical DNA lesions and the kinetics of DSB repair were followed by immunofluorescent detection of histone variant H2A.X phosphorylation at serine-139, and quantified in MSCs following treatment with conditioned media conditioned derived from NCI (**A**) or A549 (**B**) NSCLC cells by counting the amount of respective H2A.X foci per nuclei at the indicated time points

following RT with 5 Gy (0,5, 3 hours) and 10Gy (24 hours). Data show means ± SD from n=4 independent experiments (with at least 20 cells per condition and per independent experiment each). P by two-way ANOVA, followed by post hoc Sidak's multiple comparisons test: $^{**}P \leq 0.01$ and additionally by unpaired (two-tailed) t tests depicted as # $p \leq 0.05$. (**C**) Representative photomicrographs out of 4 independent experiments are shown. H2A.X phosphorylation staining are depicted in purple and nuclei were stained with Hoechst 33342 (blue) (magnification: 630x).

SupplementaryFigure S3

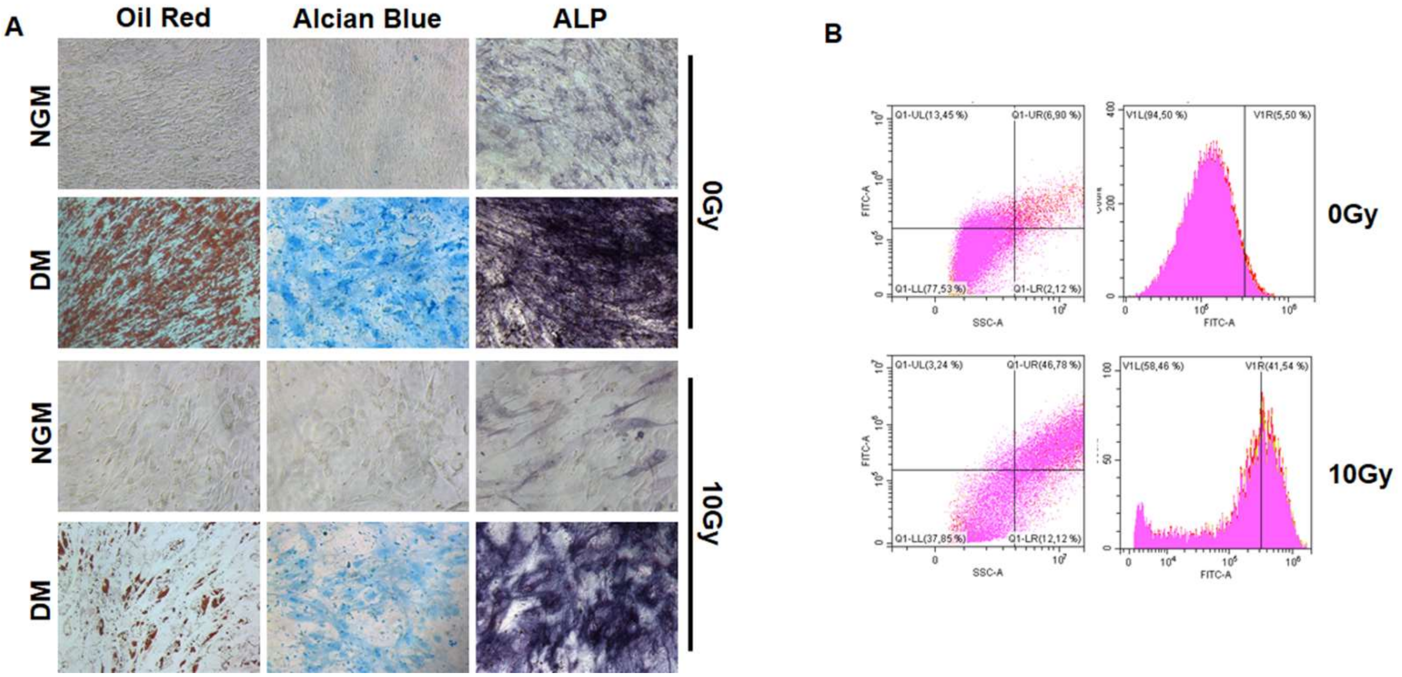

Supplementary Figure S3: RT treatment reduces the differentiation capabilities of VW-MSCs. (**A**) Trilineage differentiation. Isolated and cultured VW-MSCs were differentiated into adipocytes, osteocytes and chondrocytes following RT treatment with 0Gy (Ctrl) or 10Gy. Differentiation was observed within 14 days after induction of differentiation with differentiation media (DM) as shown by Oil red staining for detecting lipid droplets (red) in adipocytes, by Alcian Blue staining (blue) for detecting acidic polysaccharides such as glycosaminoglycans in (e.g. the cartilage-specific proteoglycan aggrecan) in chondrocytes, or by histochemical NBT/BCIP staining for detecting alkaline phosphatase activity (ALP, black-purple) in osteocytes. As control, respective cells were cultured in normal growth media (NGM). Representative photographs are shown (biological replicates: n=3-4). Magnification 400x. (**B**) Senescence (C12FDG) detection via flow cytometry analysis: (A) Representative examples of flow cytometry measurements obtained at 96 hours for control (0Gy) and 10Gy irradiations of MSCs were exemplarily shown.

Supplementary Figure S4

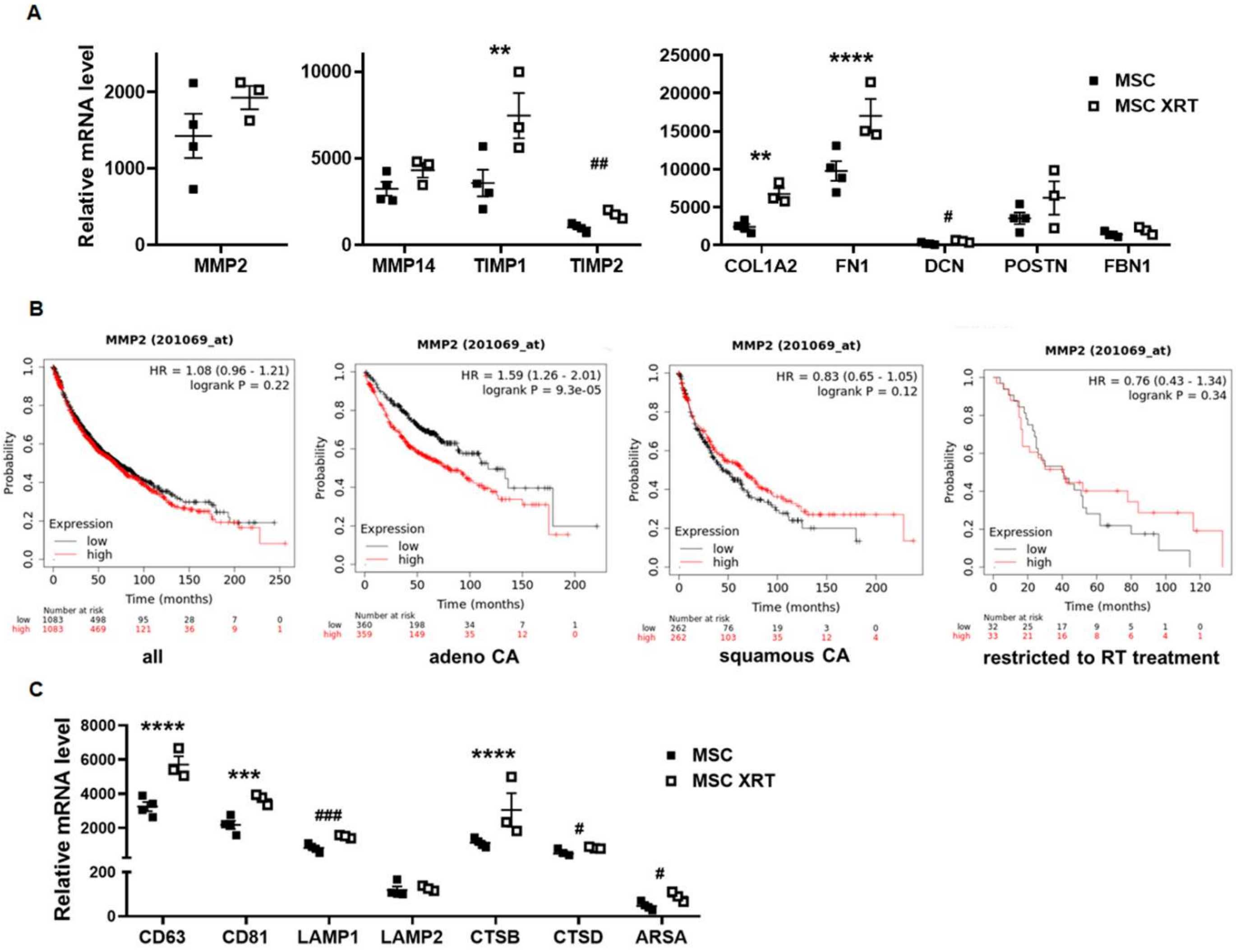

Supplementary Figure S4: The potential tumor-promoting and RT-resistance mediating role of the SASP factor MMP2. (**A**) Relative mRNA expression levels obtained from the RNAseq profiles of indicated genes (MMP2-MMP14-TIMP2 axis, as well as extracellular matrix components) are depicted. Data are shown as means ± SEM (biological replicates: MSC 0Gy n=4; MSC 10Gy n=3). P by two-way ANOVA, followed by post hoc Tukey's multiple comparisons test: **P ≤ 0.01; ****P ≤ 0.001 and additionally by unpaired (two-tailed) t tests depicted as # p ≤ 0.05; ## p ≤ 0.01. (**B**) Survival curves concerning MMP2 gene expressions were plotted for all NSCLC patients (n=2166), adenocarcinoma (adeno CA) patients (n=719), and squamous cell carcinoma (squamous CA) patients (n= 524), as well as for all available patients treated with RT (n=65). Expressions in cancer tissues above the median are indicated in red line, and expressions below the median in are summarized in black line. HR, hazard ratio. (**C**) Relative mRNA expression levels obtained from the RNAseq profiles of indicated genes (extracellular vesicles and lysosomal exocytosis genes) are depicted. Data are shown as means ± SEM (biological replicates: MSC 0Gy n=4; MSC 10Gy n=3). P by two-way ANOVA, followed by post hoc Tukey's multiple comparisons test: ***p ≤ 0.005; ****P ≤ 0.001 and additionally by unpaired (two-tailed) t tests depicted as # p ≤ 0.05; ### p ≤ 0.005.

Supplementary Figure S5

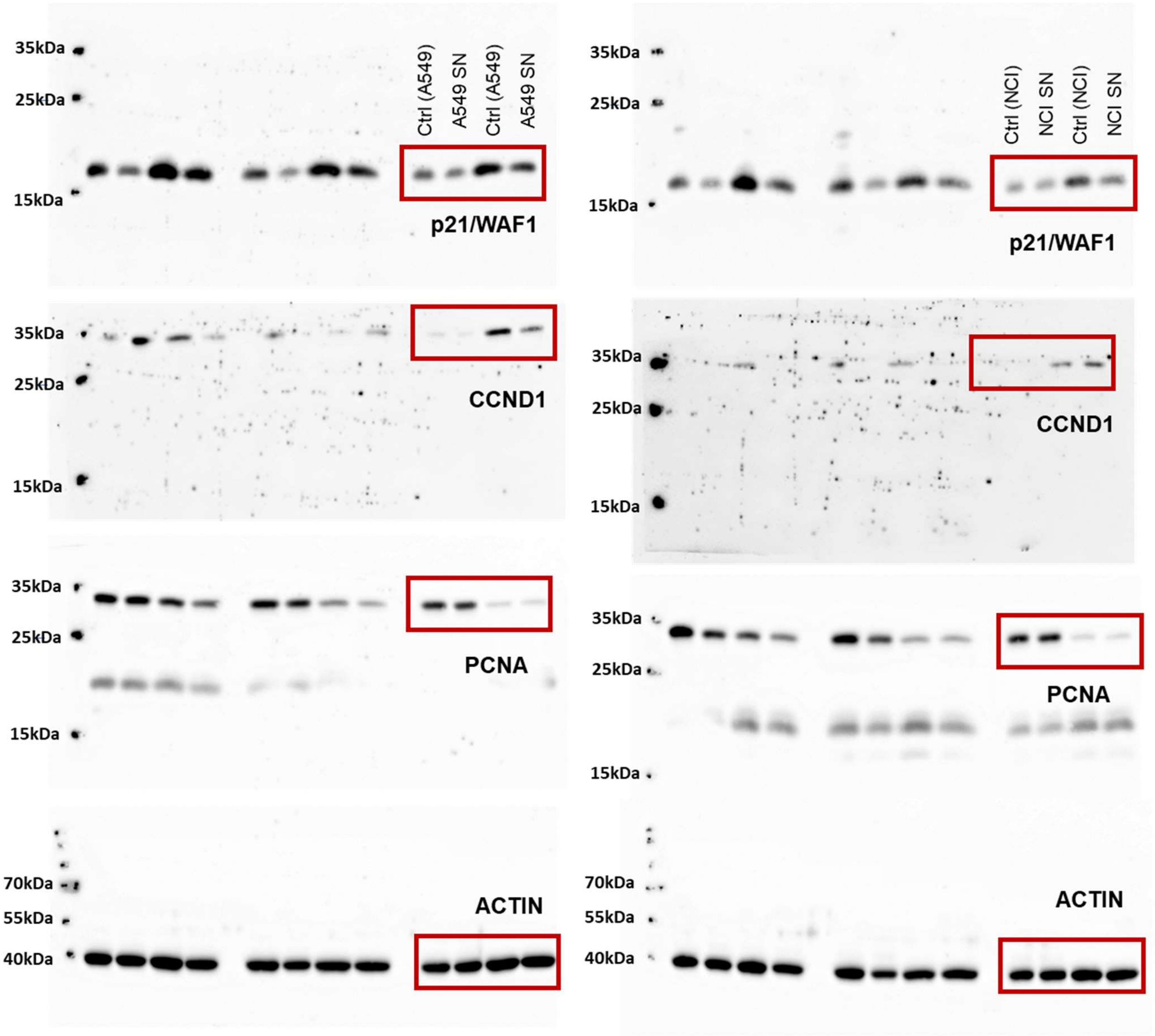

Supplementary Figure S5: Full gels of cropped gels (emphasized by a red rectangle) as shown in Figure 2H. Equal protein amounts were loaded. Beta-actin was included as a loading control.

Supplementary Figure S6

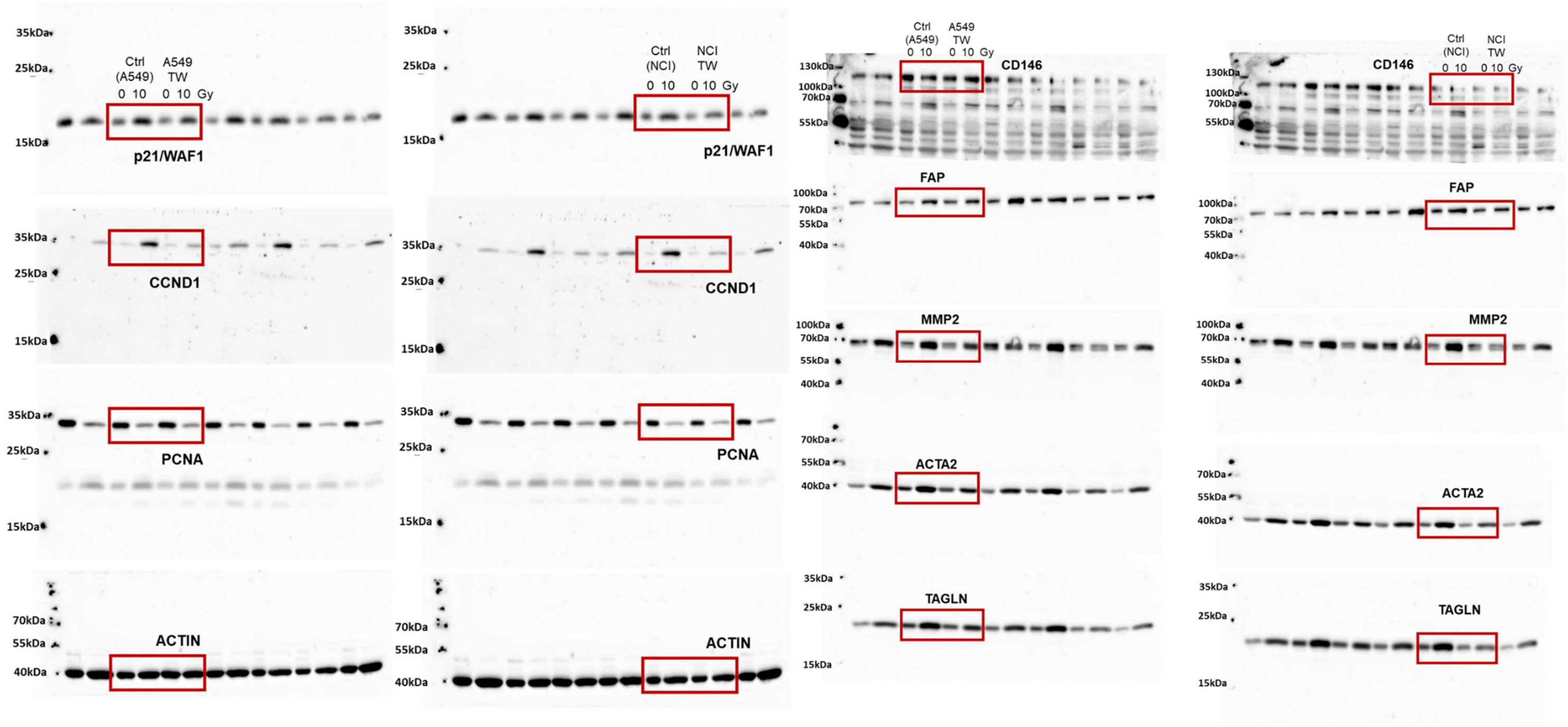

Supplementary Figure S5: Full gels of cropped gels (emphasized by a red rectangle) as shown in Figure 3D. Equal protein amounts were loaded. Beta-actin was included as a loading control.
